# Nanoparticle-Hydrogel Composites: From Molecular Interactions to Macroscopic Behavior

**DOI:** 10.3390/polym11020275

**Published:** 2019-02-06

**Authors:** Corinna Dannert, Bjørn Torger Stokke, Rita S. Dias

**Affiliations:** Department of Physics, NTNU- Norwegian University of Science and Technology, NO-7491 Trondheim, Norway; corinnad@stud.ntnu.no (C.D.); Bjorn.Stokke@ntnu.no (B.T.S.)

**Keywords:** hybrid hydrogels, nanoparticles, nanosheets, clays, polymers, adhesion

## Abstract

Hydrogels are materials used in a variety of applications, ranging from tissue engineering to drug delivery. The incorporation of nanoparticles to yield composite hydrogels has gained substantial momentum over the years since these afford tailor-making and extend material mechanical properties far beyond those achievable through molecular design of the network component. Here, we review different procedures that have been used to integrate nanoparticles into hydrogels; the types of interactions acting between polymers and nanoparticles; and how these underpin the improved mechanical and optical properties of the gels, including the self-healing ability of these composite gels, as well as serving as the basis for future development. In a less explored approach, hydrogels have been used as dispersants of nanomaterials, allowing a larger exposure of the surface of the nanomaterial and thus a better performance in catalytic and sensor applications. Furthermore, the reporting capacity of integrated nanoparticles in hydrogels to assess hydrogel properties, such as equilibrium swelling and elasticity, is highlighted.

## 1. Introduction

Hydrogels are three-dimensional cross-linked polymer networks that absorb a large amount of water when placed in aqueous solution [1]. Hydrogels are thus soft and wet materials with characteristic features of both solids, in terms of well-defined shapes, and liquids, in the large amount of free water and potentially soluble molecules that can diffuse in and out of the gels. Based on the possibility to tune the properties of hydrogels over a wide parameter space and the fact that a broad range of polymer constituents are available, these soft materials have been developed for applications such as super-absorbent polymers, contact lenses, tissue engineering, biotechnological devices, and drug delivery systems [2,3,4,5,6,7,8,9,10,11]. Biomedical applications have been particularly powered by the possibility of synthesizing polymer networks with swelling/deswelling properties sensitive to stimuli such as temperature, pH, light, or specific biomarkers. 

Large efforts have been dedicated to improving the mechanical properties beyond those of one component hydrogel networks. One such strategy is (i) the synthesis of topological gels where the network strands are interlocked by topological restrictions as realized by a slide-ring (e.g., figure-of-eight cross-links) maintaining proximity of the network strands [12,13,14,15]. These slide-ring, topological hydrogels are distinct from chemical gels cross-linked by covalent bonds between polymer chains, or physical gels where the network is established by intermolecular interactions, such as Coulomb forces or hydrophobic interactions. Other strategies comprise (ii) the development of double-network hydrogels [16] and (iii) the addition of clays and nanoparticles (NPs) to polymer solutions that act (at least partially) as cross-linkers [17]. The latter strategy, in particular, allows the formation of composite gels that are mechanically stronger due to the integration of entities supporting dissipative mechanisms [18], but also shows self-healing properties [19]. 

The incorporation of NPs into hydrogels has also been explored for purposes other than tailor-making and improving the mechanical and optical properties of hydrogels. For example, hydrogel particles can be made magnetic by the incorporation of magnetic NPs, thus facilitating separation and recycling processes [20]; stimuli-sensitive hydrogels loaded with enzymes or NPs containing drugs have been developed for drug delivery applications [21]; and NPs can also be included in hydrogels for studying their swelling properties [22,23]. 

In this work, we briefly review different synthesis pathways that have been employed to prepare NP-hydrogel networks, and outline the impact of NPs in improving various properties of the nanocomposites as compared to conventional materials. Furthermore, we present applications that are unique to nanocomposites. As a basic reference framework for the discussion, we summarize the free energy of hydrogels, including the relevant molecular parameters. Thus, the free energy of a hydrogel Δ*F_gel_* is conventionally described in three additive contributions, reflecting deformation, Δ*F_el_*; mixing of the polymer with the solvent, Δ*F_mix_*; and a term arising from the charged nature of polymers affecting the ionic balance between the hydrogel and the immersing solution. In a simple form, these terms are given as:

(1) $$\Delta F_{el}+\Delta F_{mix}+\Delta F_{ion}=\frac{\upsilon k_{B}T}{2}\left(\lambda_{1}^{2}+\lambda_{2}^{2}+\lambda_{3}^{2}-3\right)+\frac{k_{B}T}{v_{s}}\left(v_{s}c\ln\varphi_{2}+\chi \varphi_{2}\right)+\Delta F_{ion}$$

where υ is the molar number of elastic active polymer chains in the gel; $k_{B}$ is the Boltzmann constant; *T* is the absolute temperature; $\lambda_{I}$, with *I* = 1, 2, and 3, represents the deformation ratio in the three orthogonal axis directions; $v_{s}$ is the volume per solvent molecule; *c* is the nominal solvent concentration (number of solvent molecules per unit volume of the polymer); $\varphi_{2}$ is the volume fraction of the polymer phase; and χ is the Flory-Huggins interaction parameter. There are also extensions of this expression which include a more detailed account of reference states, as detailed in Ref. [24]. Equation (1) provides a basis for linking the understanding of mechanical properties (e.g., deformation at a constant swelling volume), such as shear modulus *G* defined from the proportionality between the shear stress *t* and shear *γ*:

(2) $$\frac{d\Delta F_{el}}{d\gamma }=G\gamma ;\;\mathrm{where}\;\gamma =\lambda_{1}-\lambda_{1}^{-1}$$

Calculating *G* from Equation (1) yields $G=\upsilon k_{B}T$, thus providing a direct interpretation of how changes in υ, as can be experienced in NP–hydrogel networks, affect the mechanical properties. Equation (1) also provides the basis for the interpretation of how molecular parameters affect the swelling equilibrium of hydrogel (e.g., minimum free energy as adjusted through changes in *φ*_2_ and stretching of the network chains). Conventionally, the swelling pressure is used for the description of free hydrogel swelling equilibrium, where the last term gives rise to the osmotic pressure term representing the Donnan equilibrium. This term has also been elaborated to include molecular parameters of the network and the valence of the electrolytes, as described in Ref. [25]. A recent review by Creton [18] gives an overview of different networks and gels and their properties, as well as recent advances in fine tuning the behavior of these networks.

## 2. Design of Nanoparticle–Hydrogel Composites

NP-hydrogel composites can be roughly divided into two classes, depending on the dimensions of the system. 

### 2.1. Nano- and Micro-Gel Composites

**Figure 1:**
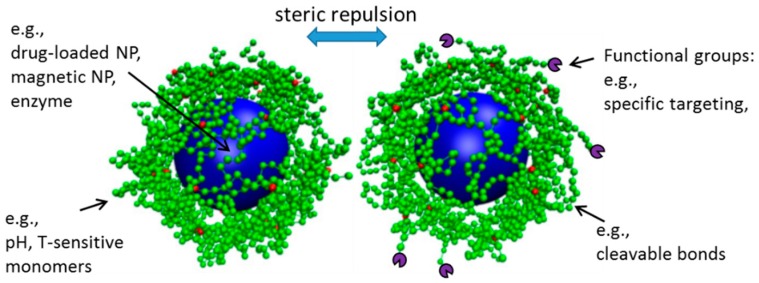
Nanoparticle—nanogel complexes depicting steric repulsions between the adsorbed polymers chains, and possible functionalization of the polymers in the nanogels. Snapshots were adapted from Nicholas Christiansen’s master thesis, August 2018, NTNU.

The first class refers to systems composed of one or a few NPs, where typically, a (small) hydrogel serves as a coat to the NP, giving a core-shell type of structure (Figure 1). Such systems have a large application potential. Nanogels can be prepared from proteins (e.g., collagen and fibrin) and other naturally-derived polymers, such as DNA, chitosan, hyaluronic acid (HA), and alginate [26,27], and have a large loading capacity for cargo ranging from inorganic NPs to biomacromolecules, such as enzymes and nucleic acids, as well as hydrophobic drugs, which makes them desirable for drug delivery applications [28,29,30,31]. As depicted in Figure 1, the soft polymer coat that surrounds the NP (cargo) makes these systems less likely to aggregate, due to the steric repulsions between particles, and provides protection to the encapsulated biological molecules from degradation and elimination, assuring a longer circulation time of active biological content [29,32]. Nanogels prepared from stimuli responsive polymers can be used for controlled delivery of the cargo, by exploring their swelling properties [1]. For example, the pH-triggered swelling of nanogels has been suggested to improve the uptake of nuclear acting drugs (chemotherapy) via the endocytic pathway due to the swelling of the nanogels in the acidic lysosomal compartments and consequent release of the drugs. These ultimately diffuse into the cell nucleus, which was not observed to occur using nanogels that do not possess the pH-sensitive moiety [33]. In a different and elegant design, the triggered release of the cargo was achieved by using gels with bonds that are either easily hydrolysable or cleaved by ubiquitous enzymes like esterases, or by enzymes that are over-expressed in the target cells [34]. Temperature, light, and magnetic-responsive nanogel composites have been explored for applications within drug delivery, as soft robots, sensors, and vehicles for capture and delivery [35]. Magnetic-responsive nanogel composites in particular are also popular in imaging and diagnostics, as reviewed in [36]. Systems composed of small dimension gels surrounding an NP core can also be used as an intermediate step in the formation of hydrogel shells with liquid cores, achieved by the dissolution of the NP core [37]. 

The approaches reported so far within this class of materials are valuable, but at the same time mostly of an empirical character that can be exploited as a qualitative basis for design guidelines. Further progress to a priori support the design of NP-loaded hydrogels with a predetermined drug loading capacity (given e.g., basic information on hydrophilic/hydrophobic nature), and, e.g., tailor-made circulation time, is currently awaiting.

### 2.2. Macroscopic Hydrogel Composites

The second class of NP–hydrogel composites gathers larger hydrogel entities, e.g., µm–cm dimensions, where NPs are incorporated at various concentrations, depending on the particular enhancement of functionality or application. Similar to the micro-/nanogel class, the macroscopic hydrogel composites can be classified according to the dominating type of interaction of the NP with the network chains, e.g., covalent or non-covalent, as well as their combination with polymer networks being either chemical, physical, or topological.

**Figure 2:**
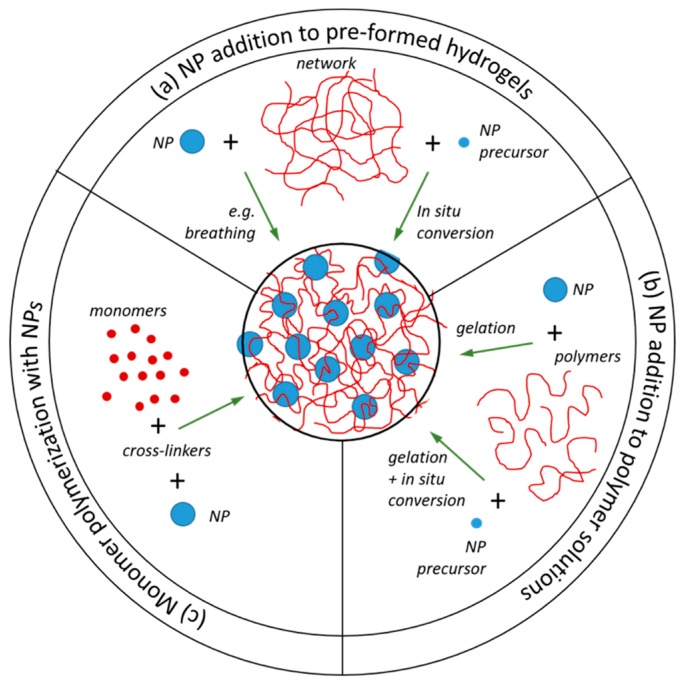
Approaches used for preparing nanoparticle—hydrogel composites: (**a**) NP addition to pre-formed hydrogels; (**b**) NP addition to polymer solutions and (**c**) Monomer polymerization in the presence of NPs. See text for details.

Independently of the class, the incorporation of NPs in hydrogels can be achieved by applying different routes. NPs can be added to: (a) pre-formed hydrogels; (b) polymers solutions that are subsequently gelled, either using the NPs as cross-linkers or independently of their presence; or (c) monomer solutions prior to co-cross-linking polymerization, in the presence of the NPs. It is also possible to grow the NPs from precursors that are incorporated in the polymer network using strategies (a) and (b) (Figure 2). 

### 2.3. Nanoparticle Addition to Pre-Formed Hydrogels

NP addition to pre-formed gels can be further divided into the synthesis of NPs using the polymer networks as scaffolds (in situ conversion) and the addition of pre-formed NPs to hydrogels (Figure 2a). The former has been particularly popular for the synthesis of metal NP-doped microgels for applications such as catalysts [38], biosensors [39], engineering optical devices materials and device architectures [40], the development of antimicrobial materials [41], and other biomedical applications [42]. The microgel structure can, in principle, be used for growing an NP in each of the polymer voids (whose size can thus be controlled by the cross-linking density). Components other than the NPs can also be loaded in the gels (such as drugs or small proteins), in which case the NPs can be used, e.g., to control drug burst release [43]. In addition, the composition of the polymer network can be explored so that the microgel is responsive to stimuli like temperature, light, or pH [44]. The methodology was first described by Antonietti and co-authors, where gold and iron oxide colloids with “non-classical shapes” were synthesized within polystyrene sulfonate microgels. Three typical morphologies were obtained, depending on the reduction rate and reducing agent: small and spherical colloids, long threads, and “nanonuggets” (algae-like). The shape of the NPs could be further controlled by the cross-linking density of the gels, where a larger cross-linking density resulted in thinner structures and more spherical colloids. These systems are based on the electrostatic attractions between the metal ions and the oppositely charged polymers in the network. Better control of the NP density within the gel can be achieved by incorporating chemical groups in the polymer network that have affinity to the NP precursors (nucleation groups). It has been shown that it is possible to control the size, morphology, and stability of the NPs by adjusting the concentration of the nucleation groups in the hydrogel side chains [45]. 

Pardo-Yissar and co-authors explored the cyclic deswelling and swelling of polymer networks in a poor and good solvent, acetone and aqueous suspension of NPs, respectively, to incorporate pre-formed Au NPs on polyacrylamide films, in what they name a “breathing” mechanism [46]. Such a procedure was chosen since the polymer film could not be formed in the presence of the NPs, due to NP aggregation. It is suggested that during the breathing out cycle, when the gels are placed in acetone and de-swell, the NPs remain inside the gels due to the collapse and physical entanglements of the polymer chains and hydrogen bonding between the monomers and the citrate surface of the NPs. The authors reported an increased NP concentration inside the gel with each breathing cycle and that the NPs were well-distributed within the film [46]. However, it was not clear if such systems are stable in aqueous solution, where NP leaching from the hydrogel may occur with time. A different procedure was adopted to co-assemble colloidal Au with poly(*N*-isopropylacrylamide) (PNIPAm) microgel, based on the centrifugation of the microgel particles with the NPs, followed by removal of the supernatant and redispersion of the Au particles through the microgel pellet by gentle heating, agitation, and sonication cycles [47]. The resulting composite could be further manipulated by light exposure where the Au plasmon absorption was explored to induce local heating, thus triggering local deswelling within the PNIPAm microgels. 

Albeit apparently working, further fundamental insight on the breathing process for the incorporation of NPs can potentially be obtained by determination of the mesh size of the network at various conditions of cycling, and how the mesh size is controlled by the molecular parameters. Such a strategy could provide more fundamental knowledge to the design of NP–hydrogel composites with respect to adjustment of the size of NPs relative to the mesh size of the network, and thereby also control the efficiency of the incorporation process, as well as the leaching of NPs.

### 2.4. NP Addition to Polymer Solutions Prior to Network Formation

**Figure 3:**
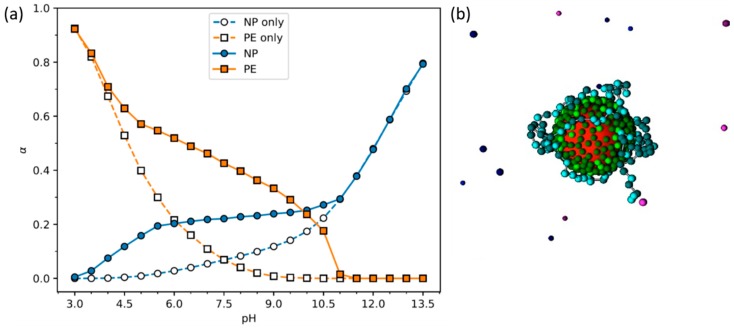
(**a**) Titration curves for a 120-monomer long polyelectrolyte (PE) (orange) and NP (blue) with pH sensitive groups both separately (dashed lines) and in a mixture (solid lines), given as the fractional charge (α) as a function of pH, obtained from Monte Carlo simulations. The difference between the dashed and solid lines shows that the fractional charge of the individual components increases in the presence of the oppositely charged NP or polymer. (**b**) Representative snapshot of adsorbed PE onto the NP. The PE is described as a sequence of hard-spheres (in green) connected by harmonic bonds. The NP is modeled as a hard-sphere with 20 Å with a density of 4.8 surface sites per nm^2^ (in blue). Dark monomers and surface groups correspond to neutral groups, and particles with bright colors refer to charged groups. The pKa of the monomers and NP surface groups was set to 7.0 and 6.8, respectively. Purple and black particles are the counterions of the PE and NP, respectively. For details on the calculation method and model, see Refs [49,50]. Data and figures by Morten Stornes.

The synthesis routes described so far, in situ formation of NPs in a preformed hydrogel network and mixing pre-synthesized NPs into polymeric networks, are widely employed for the preparation of iron oxide NP—hydrogel composites. Other strategies comprise the blending of pre-formed NPs with polymeric chains prior to cross-linking of the polymer chains, or mixing the polymer chains with iron salts for the formation of both NPs and a network during the cross-linking step (Figure 2b). Within the former, different procedures have been developed. One can simply exploit the addition of NPs to polymer solutions, followed by polymer gelation. In Ref. [20], for example, chitosan/poly(vinyl alcohol) (PVA)/Fe_3_O_4_ hybrid gel particles were prepared using an instantaneous gelation method where aqueous solutions of the three components were first mixed, followed by dripping of the solution through a needle into a sodium hydroxide bath. Such hybrid gel particles were developed as low-cost recyclable biomaterials for the removal of dyes from aqueous solution, where the inclusion of the magnetic NPs allows the separation and recycling of the gel particles using magnetic fields. The mechanism with which the NPs are kept within the gel was not elucidated in the work. Liu and co-workers explored variations in pH to assemble polyhedral oligomeric silsesquioxane (POSS)/carboxymethyl cellulose (CMC) hybrid hydrogels [48]. They started with the NPs in a solution at high pH, where the surface NH_2_ groups were not protonated, to avoid electrostatic interactions between the particles and the COO^−^ groups in CMC and the concomitant formation of a precipitate. After mixing the POSS with the CMC, the hydrolysis of added D-(+)-gluconic acid δ-lactone slowly reduced the pH, which, according to the authors, induced the formation of the hybrid network through the formation of hydrogen bonds between POSS-NH_2_ and CMC. It is unclear if hydrogen bonds are indeed the driving force for the association of CMC with POSS-NH_2_ (see discussion in Section 3). The presence of charged COO^−^ groups in the polymer chains is expected to facilitate the protonation of the NH_2_ groups in the POSS and vice-versa, as the pH is decreased. Indeed, the pKa of such groups has been found to change significantly in the presence of oppositely charged macromolecules, and can possess a charge for wider ranges of pH values than the pure substances (Figure 3) [49]. Recent work instead suggests that the formation of a polymer network may still be due to electrostatic interactions between the CMC and the POSS NPs, but by starting with weakly charged NPs and changing the pH so they slowly obtain more charge, the polymer chains will likely interact with and bridge several NPs, creating a network, instead of strongly interacting with one (or few) NP(s), which leads to the formation of neutralized large complexes (precipitate) [50].

Poly(sodium acrylate) (PANa) grafted with monomeric groups with a low critical solution temperature behavior in water (N-isopropylacrylamide, NIPAm) was mixed with SiO_2_ NPs to form hybrid networks [51]. Despite the electrostatic repulsions between the PANa backbone and the silica NPs, the formation of hybrid networks with viscoelastic properties comparable to those of covalently cross-linked gels was observed. It was proposed that the NIPAm side chains interact specifically with the silica NPs, via the formation of hydrogen bonds between the alkylene oxide units and the silanol groups, respectively, with the NPs serving as cross-links of the polymer network for the NIPAm grafted PANa. PANa chains grafted with poly(ethylene oxide-co-propylene oxide) (PPO) side chains did not show the same interaction strength with the NPs. 

It is challenging to prepare homogeneous hybrid gels with large polymer fractions simply by blending the components if the interactions between the polymer and NPs are too strong, as these tend to associate strongly, leading to heterogenous materials [52]. In such cases, a viable approach for the synthesis of hydrogel NP composites is polymerization of the polymer network, from the monomers, in the presence of the NPs, with or without a chemical cross-linker (see Section 2.5).

In a different strategy, Pasqi and co-authors describe the synthesis of composite hydrogels containing CMC polymer chains and TiO_2_ NPs functionalized with amine groups, thus supporting covalent binding of the NPs to the hydrogel network. The NPs served as cross-linkers in the hydrogel, allowing for the mechanical advantages of hybrid gels, but without some of the disadvantages, such as the leaching of NPs from the network, a common problem in hybrid networks where the NPs are only physically embedded in the polymer network, making these ideal to applications within, for example, tissue engineering [53]. A similar procedure has been followed to synthesize gelatin/Ag [54], pectin/hydroxyethyl methacrylate/TiO_2_ [55], and chitosan/Au [56] hydrogel composites, among others. In addition to the increased stability of the formulation, with respect to the release of NPs from the networks, such “click-hydrogels”, where the NPs are covalently bond to the polymer network, have the advantage of multivalency, where many polymer chains can be linked to a single NP, which increases factor ν in Equation (1), as opposed to the most common tetra-functionality of the cross-links in chemical gels. On the other hand, the presence of covalent bonds between the NPs and the polymer network restricts, to a large extent, the extensibility and self-healing potential of the hybrid material, when compared to hybrid hydrogels where the NPs are not covalently bound to the polymer network. 

As mentioned, it is also possible to mix metal salts and polymer solutions for the simultaneous formation of NPs and cross-links within the hydrogel matrix. This is described as the best method to obtain monodisperse NPs and a good colloidal stability, while exploring the advantage of a one-pot strategy [57]. This methodology has been used to produce hybrid gels with different compositions, e.g., PVA/PNIPAm/Fe_3_O_4_ obtained upon the mixing of PVA (or PVA and PNIPAm) polymers with iron salts, followed by the drop-wise addition of this solution into an ammonia solution [58], or polysaccharide-grafted poly(*N*,*N*-dimethylacrylamide) (PDMAm)/iron oxide particles, prepared using a similar procedure [57].

### 2.5. Monomer Polymerization in the Presence of Nanoparticles

The polymerization of monomers into a network in the presence of pre-made NPs (schematically depicted in Figure 2c) is probably the most common procedure to incorporate NPs into polymer networks. This strategy was pioneered by Haraguchi and co-workers [17,19,59], using a free radical polymerization of NIPAm or *N*,*N*-dimethylacrylamide (DMAm) in the presence of clays. It is suggested that the polymerization starts at the (large) surface of the clay (diameter of 20 to 30 nm and thickness of 1 nm), evolves throughout the solution, and leads to a network where the clay particles act as cross-links [60]. These gels are physical composite hydrogels due to the nature of the interaction between the polymers and NP and the lack of an added cross-linker. This methodology has been extended to prepare composite hydrogels made of modified clay particles or of spherical silica or titanium oxide NPs, both in the presence and absence of cross-linker agents, with the intention of improving the mechanical properties of the gels. Many of these examples can be found listed in Section 4.

In addition to inorganic clay and metal nanoparticles, this approach has also been explored in the preparation of hydrogel composites containing metal-oxide nanometer-scale sheets (nanosheets, NSs). As recently reviewed [61], NSs are two-dimensional nanomaterials possessing very large aspect ratios, and a range of unique chemical, physical, electronic, and optical properties. Of special interest are NSs such as titanium oxide or graphitic carbon nitride (g-CN), which, in addition to serving as reinforcing fillers and introducing function in the hydrogels (see Section 5.4), can trigger the in situ radical polymerization of acrylamide monomers and derivatives, by radiation with ultraviolet light and the concomitant production of hydroxyl radicals [62]. Such photo-initiated free-radical polymerization leads to the formation of hydrogels even in the absence of cross-linkers, but adding cross-linkers significantly increases the mechanical strength of the gels [63]. 

In an example involving biological polymers and nanomaterials, Serizawa and co-workers have found that macromolecular crowding induced by water-soluble polymers allows the enzymatic synthesis of cellulose-based nanoribbon hydrogels and suppression of cellulose NS formation and concomitant precipitation [64]. Such suppression is believed to be due to an increase in the effective viscosity of the synthesis media and/or depletion stabilization of the colloidal products [64,65]. In a recent publication [65], the same group extended this concept to form nanocomposite hydrogels by polymerizing cellulose-derived monomers, with cellulose nanocrystals (CNCs) acting as crowding agents. A reported self-assembled peptide/g-CN hydrogel, developed as a light-responsive scaffold material, is an interesting example of a nanocomposite hydrogel where non-covalent interactions are explored to both polymerize the peptide monomers (monomer—monomer interactions) and induce cross-link formation (monomer—NSs interactions) [66].

## 3. Overall Mechanism and Forces Involved in Hybrid Composite Formation

NP—hydrogel systems have received much interest in the past decades, fueled by recent novel applications (see Section 5 and Section 6). The interactions between the polymer network and the NP surface have not been studied to the same extent and, in many of the systems, are poorly understood. Considering that the improvement of the mechanical properties of the gel systems arises from the interaction of these with the surface of the NPs, understanding the interactions at play in these systems is fundamental for tailor-making such materials.

The general consensus is that NPs act as multifunctional cross-linkers for the polymer network; that is, two or more polymer chains in the network bind to one NP, and the polymer chains, in turn, bridge two or more NPs. The formation of composite networks necessarily relies on the affinity between the polymer monomers and the surface of the NPs. The adsorption of the monomers should be at least comparable to the thermal energy (~$k_{B}T$), since the polymer loses conformational entropy upon adsorption to the particle surface. On the other hand, the interaction strength between the polymer monomers and the NP surface does not need to be very large: the ability of the polymer to form multiple links, due to its conformational entropy and thus ability to adopt different conformations, dictates a strong enough interaction to restrict the infinite swelling (dissolution) of the network when placed in excess water, or the gluing of two gels mediated by NPs [67]. This concept has been further expanded by using polymer chains with dendritic end groups possessing positively charged groups. Both the large number of adhesion points and the amine groups guarantee a strong interaction with negatively charged clay surfaces [68]. Block polymers with positively charged end blocks also contribute to the formation of strong clay-hydrogel composites [69]. This mechanism underpins the improved mechanical qualities of the composite gels, described in Section 4. In addition, when the gel is under tension, some monomers and/or polymers will desorb from the NPs, dissipating the tension and retarding failure. Furthermore, such breaking in the cross-linking is reversible since the same or other neighboring strands can in turn adsorb to the NPs, replacing the detached link [70]. This mechanism also supports the self-healing capability that numerous composite NP-hydrogels show. 

Gluing hydrogels and tissue using NPs is interesting from an application point of view, but also allows a direct assessment of the adhesion forces between the hydrogels mediated by the particles. Indications of the importance of the interactions between the polymer network and the NPs arise from studies where adhesion forces between PDMAm hydrogels glued using colloidal mesoporous silica NPs were found to increase when increasing the specific surface area of the particles, achieved by increasing the pore diameter and volume of the synthesized particles [71]. Further, the particles with the largest pore diameter could absorb the largest amount of polymer at a given concentration. Additionally, it was also shown that the amount of adsorbed polymer on NPs (suspended in aqueous solution) correlated well with the gel-gel adhesion energy. Interestingly, the zeta potential of the particles decreased (from −18.5 to −35.7 mV) when increasing the specific surface area (from 219 to 514 m^2^/g), but this fact was not explored or discussed in the work [71].

Another indication that the macroscopic gel—gel adhesion is mediated by polymer—particle adsorption is based on studies where the cross-linking density of the glued hydrogels is varied. A larger concentration of cross-links leads to more constrained polymer chains and weaker adsorption energies. Furthermore, more swollen networks show weaker adhesion [67]. This is also supported by models that suggest that the energy landscape of the system is strongly dependent on the elastic properties of the gels [72]. These studies further indicate that NP dispersions can be used to assemble hydrogels, without affecting the rigidity of the material [67,72,73].

The chemistry of the NP surface and the composition of the hydrogel are important parameters in the adhesion of NPs to the polymer network and can be explored to increase the strength of the interaction and thus adhesion. One possibility is to include moieties supporting electrostatic interactions between NPs and the network. Such interactions are long ranged and the possibility of tuning their strength with variations in ionic strength and pH makes this a popular route in some applications [74,75]. The adsorption of neutral polymers onto surfaces, on the other hand, often relies on specific and short-range interactions. Poly(ethylene oxide) (PEO), for example, is a commercial polymer that can be used as a dispersant for latex and a flocculant for clays and silica particles. In fact, the ability of polymers to stabilize colloidal suspensions against flocculation, which is of large importance in the formulation of, for example, paints and cosmetics, has fueled the study of polymer adsorption onto particles and surfaces since the 50s [76,77,78].

Clays and silica NPs are recurrent compounds in nanocomposite hydrogels and it was established early on that PNIPAm and PDMAm showed relatively strong interactions with the NPs (and thus a good adsorption ability), while PVA and PAAm did not [79,80,81]. It is commonly described that hydrogen bonds established between the polymer moieties and the silanol (Si–OH) groups on the surface of the silica particles are the driving force of the adsorption of neutral polymers to the surface. Indeed, it was shown, using infrared spectroscopy, that DMA forms hydrogen bonds with the surface groups on the silica surface in the absence of water (in chloroform), which is to be expected [79]. Nevertheless, and as stated by Rubio and Kitchener, “In an aqueous medium, however, such bonding must be reduced by competition from water molecules.” [82]. These authors also mention, citing another work, that the non-adsorption of PVA could be due to the difficulty in displacing water from the silanol groups. 

**Figure 4:**
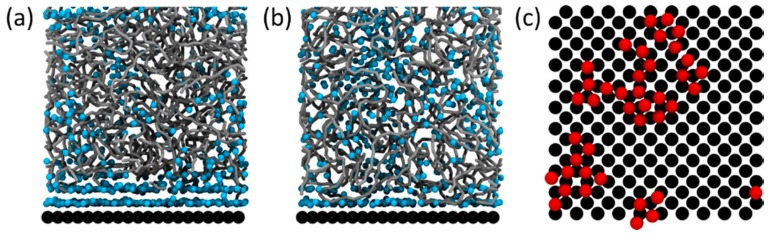
Snapshots of molecular dynamic simulations depicting solvated polymers (in gray) near a silica surface (in dark). Panels (**a**) and (**b**) refer to PAAm and PDMAm, respectively. Panel (**c**) shows the top view of the absorbed PDMAm monomers. Reprinted with permission from Perrin, E.; Schoen, M.; Coudert, F.; Boutin, A. Structure and Dynamics of Solvated Polymers near a Silica Surface: On the Different Roles Played by *Solvent Journal of Physical Chemistry B* **2018**, 122, 4573–4582. Copyright (2018) American Chemical Society.

Perrin and co-authors [80] studied the structure and dynamics of polymers near a silica surface using a coarse-grained model. The model used, based on the Martini force field, describes groups of atoms using beads (two for the monomer backbone and two for each the side chains of the monomer) with different degrees of polarity. In addition, beads can be defined as hydrogen bond donors and/or acceptors. The only non-bounded interactions included in the simulations were represented by a modified Lennard-Jones potential. The work focused on two polymers with slightly different compositions, PDMAm and PAAm, which were found to interact and not interact, respectively, with silica NPs using lap adhesion tests [67]. The interaction energy between the model silica surface and the used solvent model was found to be very slightly larger than that of silica—PDMAm and significantly larger than that of silica—PAAm side chain monomers. On the other hand, snapshots of the systems (Figure 4) show that even for PDMAm, the polymer coverage of the surface is not very large, with an apparent larger fractional occupation of solvent molecules near the surface of silica. This was especially obvious in the PAAm systems, where the polymer occupation at the surface decreased. Without discussing it much, but highlighting the fundamental role of the solvent in the absorption mechanism, Perrin and co-authors conclude that the strongest solvation of the PAAm dictates the lack of adsorption at the silica surface. These results suggest that entropic effects, rather than the direct H-bonds between polymers and particles, are the driving force for polymer adsorption onto NPs. Naturally, when the polymers do adsorb, hydrogen bonds between the monomers and surface groups can be easily formed. It is interesting to note that PDMAm, which has been shown to interact with silica NPs, has two methyl groups in each monomer, which makes it a more hydrophobic polymer than PAAm. Rubio and Kitchener studied the adsorption of high molecular weight PEO onto various forms of silica. They suggest that (i) isolated silanol groups are probably the principal adsorption sites, (ii) the adsorption of PEO is favored if the regions between the sites are hydrophobic, and (iii) the adsorption of PEO is disfavored if the regions between the adsorption sites are hydrated and (iv) particularly disfavored if the surface is appreciably ionized [82], although other work has showed no appreciable difference in PEO adsorption to silica within the pH range 2.5–10, where one would expect some variation in the ionization of the NPs [83]. Regarding point (ii), other evidence pointing to the significance of the hydrophobic interactions is the fact that a “salting-out” effect was observed in these systems; that is, the addition of 20 mM of salt led to an increase in the strength of the interaction. Conclusion (iv) arises from studies showing that the adsorption of PVA onto silica surfaces was higher at pH 3.6 than at pH 9, justified by the presence of more undissociated silanol groups to form hydrogen bonds with the polymer chains [84]. While this is a good argument, it is also suggested in Ref. [82] that the presence of ionized groups at the silica surface decreases the hydrophobicity of the surface (weakening the interaction if this is, at least partially, hydrophobic in nature), or that the presence of condensed counterions could prevent PEO from approaching the surface. 

In a recent article, an off-lattice model taking into account the excluded volume of polymers and ions is used to describe the adsorption of PVP on a silica surface. Interestingly, the predictions from the model were found to be in good agreement with experiments when both a hydrogen bonding-based interaction (based on a Lennard-Jones potential that could have been used to describe van der Waals interactions) and excluded volume effects between ions and (neutral) polymer chains were included. The effect of the latter was discussed to be two-fold: the direct competition between the polymer and the ions for surface adsorption and consequent decrease in the polymer adsorption [85], and/or an improved titration of the silanol groups enhanced by counterion condensation and, concomitantly, less opportunities for these groups to form hydrogen bonds with silica [86]. 

In a recent publication, Sato and co-authors probed the importance of van der Waals forces for the interactions between silica NPs and PAAm and PDMAm gels, by varying the Hamaker constant, *A*, achieved by changing the refractive index of the medium using ethylene glycol [87]. They observed that for sufficiently high concentrations of ethylene glycol, where PAAm and PDMAm have similar *A* values, the quantity of particles adsorbed onto each gel is similar. Hydrogen bonding and hydrophobic interactions were dismissed since the silica surface was well-hydrated and both the polymers and silica surface were hydrophilic. In their opinion, the driving force for NP adsorption on hydrogels is van der Waals interactions, at least under conditions where chemical and electrostatic adsorption are negligible.

It is clear that polymers possessing carboxyl or ether oxygen groups in their composition will form hydrogen bonds with hydroxyl groups at surfaces, and that the strength of these interactions depends on the chemistry of the groups. It is, however, not clear if this is the driving force of association or if instead van der Waals forces predominate. Additionally, entropic effects derived from a preference of the hydration waters on the particle surface and/or the polymer to be in bulk rather than associated, should not be disregarded in these systems.

While this discussion has predominantly focused on interactions between polymer network chains and clay particles or silica NPs, similar arguments can be used in equivalent systems. He and co-authors [88] describe the formation of intermolecular hydrogen bonds between g-CN NSs and sodium alginate chains, based on infra-red spectroscopy, showing a broadening and shift of the peaks corresponding to the O–H stretching vibration in g-CN NSs, and pointing out studies of sodium alginate with graphene oxides (GOs) with different surface functionalization on the formation of (dry) films [89]. Graphene NSs have attracted much interest due to their high specific surface area, electron transport, and mechanical properties [90]. As mentioned below, these materials are prone to aggregation in aqueous solution and large efforts have been devoted to increasing their stability in solution. This can be achieved by surface modifications, such as the introduction of charged surface groups, leading to stabilization through electrostatic repulsions or the adsorption of polymers and stabilization through steric repulsions. In ref. [89], it is found (not surprisingly) that (the negatively charged) alginate interacts more strongly with positively charged GOs than with GOs with modifications that introduce negatively charged surface groups. Furthermore, infrared spectroscopy was used to identify the groups involved in alginate –GO interactions, in dry films, which were attributed to intermolecular hydrogen bonds. Nanocomposite hydrogels have also been prepared using GO and PVA solutions [91]. Interestingly, the authors describe the hydrogel structure as networks of GO NSs with the PVA acting as a physical cross-linking agent, as opposed to the most commonly described polymer network with nanomaterials acting as cross-linking sites. The PVA is stated to function as a cross-linking agent since the GO NSs can interact weakly in pure solution. Additionally, in these systems, hydrogen bonding between the hydroxyl, epoxy, and carboxyl groups at the surface of the GO sheets and the hydroxyl-rich PVA chains is considered to be the dominant force, again based on the characterization of dry samples [92].

It is worth mentioning that the predominant role of hydrogen-bonding in cellulose association in water, and the interaction of cellulose with non-ionic polymers in aqueous solution, has recently been challenged [93,94,95].

As mentioned above, it is possible to make use of the photo-initiation abilities of g-CN NSs to trigger the in situ radical polymerization of acrylamide monomers and derivatives [62,96]. Since radicals can be generated at the surface of the g-CN NSs, it is suggested that polymerization starts at the surface of the NSs, which also acts as anchoring points for the monomer, i.e., the polymers are effectively covalently bound to the surface of the NSs. The formation of strong hydrogels without the addition of external cross-linkers is, according to the authors, a clear indication of the formation of covalent bonds between the NSs and the polymer network [97]. In addition, control experiments where the NSs were physically embedded in the hydrogel via redox initiation in the dark (no photo-initiation) created a hydrogel that was still stronger than the (PDMAm hydrogel) control, due to the filler reinforcement effect of the NSs, but weaker than the gels where the polymer is bound to the NSs. Still considering g-CN NSs, an interesting self-assembled peptide/g-CN hydrogel nanocomposite was recently reported where both the peptide polymerization (monomer-monomer interaction) and interaction between peptide monomers and NSs were driven by non-covalent π-π interactions between the aromatic rings in the monomers and the aromatic rings of the monomers and the graphitic structure of the NSs, respectively [66].

A recent article by Liu and co-authors [98] has highlighted the need for careful structural and mechanistic studies within the NP–hydrogel nanocomposite field. While studying the effect of the size and shape of silica NPs on biological tissue (pig liver) adhesion, the authors came across an unexpected effect of NaOH or KOH bases in the tissue gluing process. It was found that the synthesized NPs achieved tissue adhesion that was similar to that obtained using commercial Ludox silica NPs (SM-30) [67] only after exposing the NPs to a concentrated base solution. Such treatment caused the etching of the NP surface, changing the particle size and shape into “poorly defined clusters of spherical particles”, and changing the surface charge of the NPs from negative to nearly neutral [98]. Surprisingly, much stronger tissue adhesion was achieved by simply adding a drop of strong base solution to two liver surfaces that were pressed together without the addition of NPs. It is suggested that the strong base changes or damages (possibly denaturates) the surface proteins of the pig liver, which enhances protein-protein interactions and improves the adhesion of the tissue. In this respect, and according to the authors, the presence of the silica NPs is unnecessary or even a hindrance to the adhesion process.

In fact, SM-30 has been shown to glue hydrogels made of PDMAm [67], in a system where it is not clear how a strong base would affect the hydrogel–hydrogel interactions. In addition, PDMAm polymers were shown to adsorb to silica particles (including Ludox) [71]. Furthermore, Stöber silica NP solutions [73] and colloidal mesoporous silica NPs [71] have been shown to glue rat skin and enhance wound healing, apparently without the aid of a strong base.

While the proposed base-induced protein denaturation mechanism is a rather interesting one and certainly worthy of further studies, it does not seem to be the only one at play. Having in mind, in particular, the studies involving ex vivo (pig [98] and calf liver [67]) and in vivo (rat skin [73]) systems, one may also have to consider tissue composition specificity, as well as the presence of components that may only be present in living tissue, while developing NPs for tissue adhesion and discussing the relevant interactions at play.

## 4. Nanoparticles for Improving Hydrogel Properties

### 4.1. Optical Properties

Typically, gels are transparent and flexible at a low cross-linker content and become opaque and fragile at a high cross-link density [99]. This has been attributed to the development of structural inhomogeneities [100]. A decrease in transmittance can also be obtained by variations in temperature when gels are composed of temperature-responsive polymers. For example, PNIPAm polymers exhibit a coil-to-globule transition for temperatures above the lower critical solution temperature, which leads to changes in gel transmittance [59]. It is therefore important to have enough information about the behavior of the polymers at different temperatures and pHs in order to be able to establish whether the transmittance varies because of the cross-link density or other factors [101].

Assuming that gel transmittance is decreased upon the formation of heterogeneous domains, the observed larger transmittance of nanocomposite hydrogels suggests that the cross-links in nanocomposite gels are more uniformly distributed [59] and that only a very small amount of the NPs form oligomers or bigger clusters [102]. Oligomers and bigger clusters, in addition to heterogeneity, can lead to an increase in diffusive scattering, which decreases the transparency [103]. Accordingly, the optical behavior of, for example, cellulose-based hydrogels with silicate NPs, was observed to be highly dependent on the concentration of NPs in the network [103,104], which was explained by both an increase in cross-link density and an increase in NP aggregates [103]. On the other hand, Haraguchi et al. [59] reported that PNIPAm–clay nanocomposites show a constant transparency for clay NP concentrations up to 60 wt % (against polymer) (see Figure 4 in Ref. [59]), which could indicate that gel transmittance is independent of clay concentration. It is most likely, however, that all nanocomposite hydrogels show a decrease of transparency above a certain critical concentration of NPs, which was simply not reached in the study by Haraguchi and co-workers. The NP concentration where the decrease in transmittance is observed will depend on the properties of the NPs and on the intermolecular interactions between NPs and between NPs and the polymers in the gel. 

Depending on the composition, the transmittance of nanocomposite gels may also be sensitive to pH variations. For example, Ahmed and co-authors [105] showed that nanocomposite hydrogels obtained from sodium silicates/colloidal silica mixtures, developed for bacterial encapsulation, lost transparency when lowering the pH from 7 to 5 (as well as the ability to gel) when using Ludox silica particles, while sodium silicate NPs showed the opposite trend, with samples becoming more opaque when the pH was increased from 5 to 6. The authors concluded that the transparency variations were related to NP aggregation upon pH variation and that by varying the pH and NP concentration, whose increase resulted in an increase in optical density, it was possible to synthesize gels with a similar transparency for a wide range of silicate concentrations [105]. 

In addition to temperature, NP concentration, and pH, swelling and de-swelling can affect gel transmittance. Yang et al. [106] showed that some NP–hydrogel composites turned white on de-swelling, ending up at 0% transmittance. However, in general, nanocomposite hydrogels are highly translucent materials [103] and can exhibit a transmittance above 90% even for high concentrations of NPs [59,107], depending on the general composition, pH, and temperature of the solution. Overall, hydrogel composites show a higher transparency than chemically cross-linked gels due to less structural inhomogeneities that arise when chemical cross-links are formed inhomogeneously during polymerization [108].

### 4.2. Mechanical Properties

The mechanical properties of hydrogel composites can depend strongly on the concentration of NPs, but tend to behave differently from covalently cross-linked gels [52]. In addition, the length of the polymer chains also plays an important role in the mechanical properties of the hydrogels; the longer and more flexible the polymer chains between the cross-linking points (NPs) are, the greater the flexibility of the nanocomposite hydrogel [109]. This is also true for covalently cross-linked gels; however, the number of cross-links is much higher than in hydrogel composites [109]. Due to this, the chain length between cross-links is generally larger in nanocomposite gels than in covalently cross-linked gels composed of the same polymers [109]. In the following, we discuss the impact of NP integration in hydrogels on the mechanical properties of the resulting materials, with an emphasis on stress-strain behavior, including the elastic and storage moduli, and stress relaxation. 

#### 4.2.1. Stress-Strain Behavior

In contrast to covalently cross-linked hydrogels, nanocomposite hydrogels typically exhibit an improved behavior in response to stress. Multiple studies have shown that NP–hydrogel networks can withstand high degrees of deformation, such as elongation, bending, compression, and tearing [110,111,112]. 

Nanocomposite hydrogels have been shown to exhibit an increase of both shear storage modulus (*G*’) [59,68,74,103,112,113,114,115] and Young’s modulus (*E*) [52,59,102,103,105,107,109,114,115,116] in comparison to covalently cross-linked hydrogels with a similar polymer composition prepared with some form of cross-linking agent (for example *N*,*N*′-methylenebis(acrylamide)) [59,109]. However, as previously mentioned, the covalently cross-linked gels generally have a higher cross-linking density [109], which might justify, at least partially, the different behavior of the materials. It was shown that increasing the concentration of NPs can lead to an increase in shear storage modulus and Young’s modulus for low NP concentrations, followed by a plateau [59,102,103,104,105,109,111,112,117,118]. This was also the case with PAAm hydrogels reinforced with graphene NSs [119]. 

Recently, magnetic fields have been used to orient stacked titanate (IV) NSs and form anisotropic composites. These were shown to resist compressibility applied orthogonally to the oriented NSs while undergoing deformation under parallel shear forces. The resistance to compressibility in these systems arises from electrostatic repulsions between the NSs [62]. Additionally, g-CN NSs have recently become a popular inorganic hydrogel filler, where the mechanical properties are also dominated by the electrostatic repulsion between the NSs, as shown by the increase in the shear storage modulus of the hydrogels upon the decrease (more negative) of the zeta potential of the nanosheets [97].

**Figure 5:**
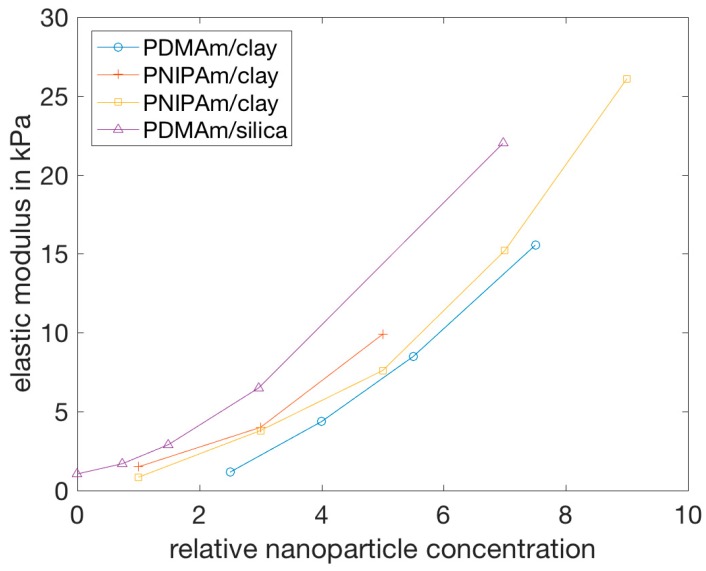
Elastic moduli for different nanocomposite hydrogels. Replotted using data from PDMAm/clay [109] (blue dots), PNIPAm/clay [17,59] (yellow squares and red crosses), and PDMAm/silica [52] (purple triangles). The concentration of clay particles is $C_{Clay}=(100)\left(\frac{m_{Clay}}{M_{Clay}}\right)$, with m_Clay_ representing the weight of clay per 1000 mL of water and M_clay_ representing the molecular mass of clay [17,59,109]. The silica NP concentration is the weight ratio between silica and DMA [52]. The amount of polymer in the samples varied from approximately 1.5 g to 6 g.

Figure 5 summarizes reported elastic moduli as a function of NP concentration for different nanocomposite hydrogels. They all show an increase of the Young’s modulus with NP concentration [17,52,59,109], even if they are composed of different polymers and/or NPs. It is important to note in Figure 5 that the definition of NP concentration is different for the silica and the clay NPs. All in all, the nanocomposite hydrogels depicted here show a comparable increase in elastic modulus. At variance, the elastic modulus of HA/silica NP hydrogels was shown to decrease above a certain NP concentration (2%), which did not occur with chemically cross-linked HA gels (using divinyl sulfone as a cross-linker) [102].

The storage modulus increases steadily as the concentration of NPs increases [59,74,113,118], showing up to a hundred-fold increase [74], until the gel reaches a critical strain region. Above this region, the storage modulus decreases rapidly, indicating that the gel collapses into a quasi-liquid state [68]. In addition, nanocomposite hydrogels can exhibit a significant strain rate dependence [49]. The important property of the nanocomposite hydrogels that leads to the increase of the storage modulus, below the critical NP concentration, is the lack of cross-links formed by covalent bonds [74].

Such response to stress indicates that:

- The NPs of the examined samples were integrated in the hydrogel networks, forming physical cross-links that cause the nanocomposite hydrogel to behave as an elastic gel with high extensibility [74,104,113];
- When the concentration of NPs increases, the resulting interactions between them become more important than the NP-polymer interactions, leading to an increase in the stiffness of the network and thus decreasing maximum elongation at break [52,103].

**Figure 6:**
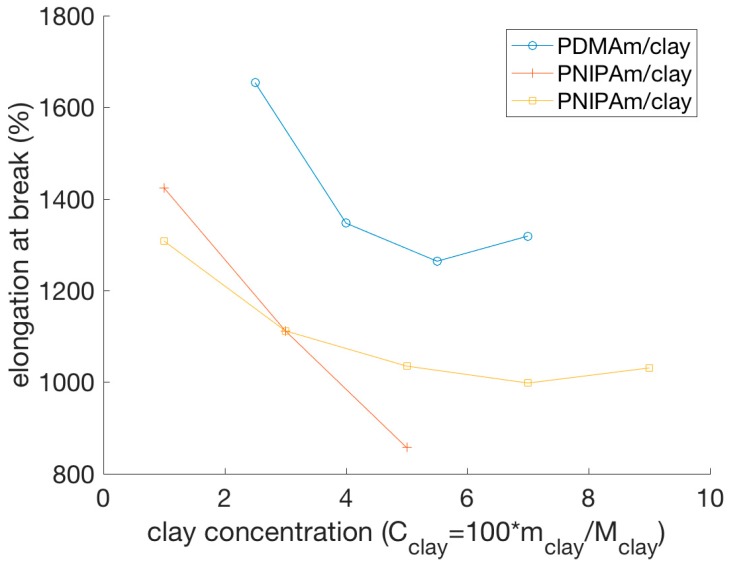
Elongation at break of nanocomposite hydrogels. Data from Haraguchi et al. [17,59,109] of elongations at break for PDMAm and clay hydrogels [109] (blue circles), PNIPAm and clay hydrogels [17] (red crosses), and PNIPAm and clay hydrogels [59] (yellow squares) replotted as a function of clay concentration C_clay_.

The elongation at break was shown to be greater for hydrogel composites than for covalently cross-linked hydrogels prepared with the same monomers and with a comparable cross-linking density [59,103,104,109,111,116,118]. While typical covalently cross-linked gels break at an elongation of approximately 50%, regardless of cross-link density [84,89], nanocomposite hydrogels could be extended to about 1000%, or more, before breaking [52,107,111,114,116], which shows an improved mechanical behavior when compared to covalently cross-linked hydrogels. Figure 6 shows the elongation at break from three different studies and clearly depicts how the elongation at break shows a tendency to decrease with increasing clay concentration, at least up to a certain NP concentration. It is unclear from the presented data points if two of the systems show a minimum in the elongation at break, or if they reach a plateau. An important observation is that the data for the PNIPAm/clay nanocomposite hydrogels, representing gels prepared (apparently) under the same conditions, do not show the same behavior, which suggests that environmental factors during the preparation or characterization of the hydrogels, such as temperature, might have an effect on the mechanical properties of the nanocomposite hydrogels. In another study, however, PVA-clay hydrogels have been shown to exhibit a drastic increase in the elongation at break as the clay concentration increased, within the studied concentrations [117]. In general, all these NP–hydrogel composites gels exhibit very high elongations at break, above 1000%, for low clay concentrations [17,59,109]. 

Some nanocomposite hydrogels can even be elongated at a sufficient length to form a knot without displaying damage or breaking in the process [59]. Also, the shape recovery after elongation can be almost 100% [59,111,112,116].

In summary, the incorporation of NPs into hydrogels can improve the elastic and storage moduli and increase the elongation at break, with the enhanced mechanical properties being attributed to the physical cross-links between NPs and polymer chains [52,104]. 

#### 4.2.2. Stress Relaxation

Nanocomposite hydrogels also exhibit a different stress relaxation than chemically cross-linked hydrogels. Following a sufficiently long waiting time after exposing a macroscopic sample of these materials to stress, the stress relaxes and reaches a plateau without further change [52]. This process strongly depends on the concentration of NPs in the gel [52], which suggests that the interactions between these and the polymers play a key role in it. This hypothesis was supported by the finding that stress relaxation also depends on the type of cross-links in the gel [101]. In nanocomposite hydrogels, stress relaxation occurs mainly through dissipative mechanisms of breaking and reforming cross-links between NPs and polymers [101], while in covalently cross-linked gels, stress relaxation occurs mainly through the migration of water [103]. Thus, the nature of NP-hydrogel interactions in nanocomposite hydrogels is the cause for this stress relaxation behavior. 

## 5. Novel Functionalities

### 5.1. Self-Healing Hydrogels

Polymer gels with self-healing abilities can regenerate, similarly to living matter, in response to damages in the polymer network and formation of cracks [120]. This repair should occur both autonomously and spontaneously [110] and can be achieved through different approaches. One of these approaches consists of using NPs as network cross-linkers. 

Table 1 shows an overview of some recent studies where self-healing nanocomposite hydrogels were synthesized and studied. 

**Figure 7:**
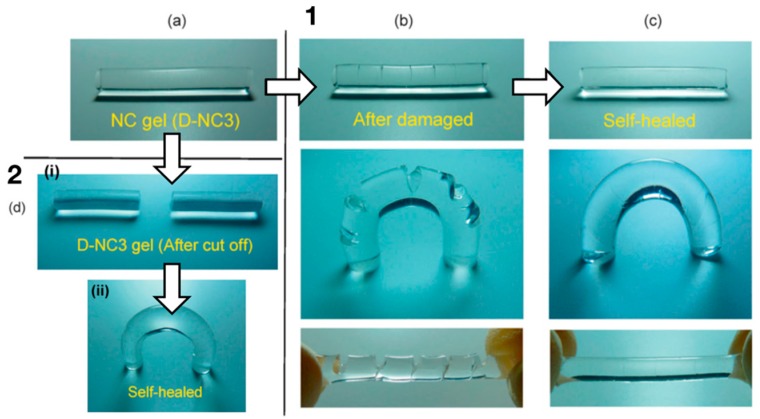
Self-healing nanocomposite hydrogel composed of PDMAm and hectorite clay NPs (D-NC3). The nanocomposite hydrogels were (1) damaged or (2) cut off using a knife and then healed by keeping the cut surfaces in contact at 37 °C for varying times, ranging from 48 to 100 h. Reprinted with permission from Haraguchi, K.; Uyama, K.; Tanimoto, H. Self-healing in Nanocomposite Hydrogels. *Macromolecular Rapid Communications* **2011**, *32*, 1253–1258. Copyright (2011) John Wiley and Sons.

Haraguchi and co-authors [110] developed a self-healing gel by synthesizing a nanocomposite gel composed of PDMAm or PNIPAm and inorganic, hydrophilic, synthetic hectorite clay NPs. Mechanical damage, i.e., knife cuts, healed autonomously, without the use of healing agents (Figure 7), just by keeping the cut surfaces in contact at ambient or slightly elevated temperatures [110]. The same behavior was also observed for other types of gels [68], suggesting it is not specific to polymer networks of a particular composition. The self-healing behavior can be attributed to the nature of the nanocomposite hydrogel. At the freshly cut surface of such a gel, long polymer chains, for example, PDMAm chains, that are adsorbed onto the surface of hydrophilic inorganic clay NPs inside the gel at one end have the ability to interact with clay particles on the other gel surface [110]. Thus, by adjoining two cut surfaces, the chains of each surface will diffuse into the network of the other surface and bridge neighboring clays, thus gluing the two gel surfaces together [110]. 

**Figure 8:**
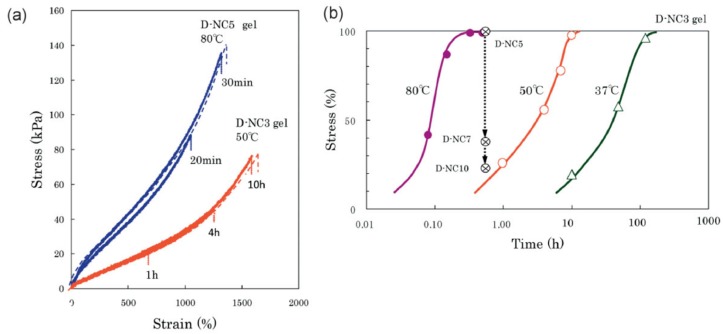
Tensile mechanical properties of self-healed PDMAm/synthetic hectorite nanocomposite hydrogels (D-NCn, where n indicates increasing concentration of hectorite) shown in Figure 7. (**a**) Stress-strain curves of the original and the self-healed gels before (dashed lines) and after (solid lines) cutting. (**b**) Effects of self-healing conditions (time and temperature) on the recovery of tensile strength of self-healed gels. Reprinted with permission from Haraguchi, K.; Uyama, K.; Tanimoto, H. Self-healing in Nanocomposite Hydrogels *Macromolecular Rapid Communications* **2011**, 32, 1253–1258. Copyright (2011) John Wiley and Sons.

For many nanocomposite gels, the time the two cut surfaces have to be in contact in order for self-healing to occur strongly depends on the temperature [107,110,118,121,122,123,124,125]. Haraguchi et al. [110] showed that at a temperature of 37 °C, the self-healing was completed, with a 100% recovery of tensile strength, after 48 h, see Figure 8. Factors that increase polymer diffusion, such as higher temperatures or a longer contact time, also increased the rate of self-healing, since the polymer chains at each surface have to diffuse into the opposite surface to create new bonds and thus glue the two surfaces together [107,110,118,125]. For example, for composite hydrogels made of PDMAm and hectorite, it took only 30 min for full self-healing at 80 °C [110]. On the other hand, self-healing hardly occurred at high temperatures for PNIPAm/hectorite hydrogels, due to the coil-globule transition of PNIPAm chains at 32 °C [110]. This shows that self-healing is dependent on the composition of the hydrogels, which needs to be considered in the development of nanocomposite hydrogels.

The ability of the gels to self-heal decreases with an increasing concentration of clay NPs (C_Clay_) [110]. In gels with very high C_Clay_, self-healing becomes very difficult due to the short lengths of the chains bridging the NPs [110]. However, if the length of the polymer chain bridging two NPs is too large, the healing time increases, since the free ends of the polymer chains need a longer time to diffuse and find another NP [125]. Self-healing did not only reestablish the tensile strength after contact for a short duration, but it was also long lasting. It could also be achieved after a relatively long waiting time after the cut, particularly for gels with long polymer chains, since long chains at the cut surfaces have a lower probability of interacting with the clay NPs within the same surface [110]. However, if the chains are short enough to bind to the same surface, the cut surfaces only adhere when they are freshly cut [68].

Nejadnik and co-authors [74] showed that self-healing does not only occur in synthetic hydrogels, but also in gels consisting of bisphosphonated hyaluronan (HABP) and calcium phosphate (CaP) NPs. In their work, it was reported that the HABP/CaP hydrogel displayed fast self-healing behavior, with the joining of two cut surfaces occurring in less than 5 s [74]. The healing occurred, as in other nanocomposite hydrogels, because the interactions between the CaP NPs and grafted bisphosphonate groups of hyaluronan are non-covalent and can thus be restored within a limited contact duration. The rapid nature of the self-healing process was attributed to the fact that the interactions (not discussed) were stronger than other less specific non-covalent interactions in other nanocomposite hydrogels [74].

Interestingly, nanocomposite hydrogels with different compositions could be combined by cutting and healing. By joining the cut surfaces of two different gels at ambient temperatures, the two gels could be combined and did not detach with alternating swelling and de-swelling treatments [110].

To summarize, it is possible for nanocomposite hydrogels consisting of long-chained polymers and inorganic NPs to show self-healing behavior. When cut, the polymers at a surface are able to diffuse into the network of the other surface and form new bonds with the NPs there. Because these bonds are non-covalent, they are easily formed and broken, which is essential for the self-healing process.

### 5.2. Adhesive Materials

Another novel functionality of nanocomposite hydrogels is related to (a few) reports on the adhesion of plastic, glass, and tissue induced by hydrogels of particular compositions [130]. It is suggested that the non-covalent interactions between the NPs and polymer chains are responsible for the adhesion and, since these can be broken and reformed autonomously, adhesion can also be reversible [130]. Wu and co-authors [131] showed that nanocomposite hydrogels consisting of poly(ethylene glycol) and synthetic silica Laponite platelets have improved adhesion properties compared to hydrogels without the Laponite NPs. The improved adhesion was attributed to the increased flexibility of the nanocomposite hydrogels, which leads to better stress dissipation [131]. Furthermore, the adhesive strength is largest for lower NP concentrations, and it decreases as the cross-linking density increases. A study by Gahawar et al. [132] showed similar results, with poly(ethylene glycol) and Laponite nanocomposite hydrogels showing the ability to adhere to osteoblast cells. This demonstrates that nanocomposite hydrogels could be used for a wide range of biomedical applications, especially in the field of wound closure [132]. Unfortunately, Laponite platelets have been shown to be cytotoxic, even at low concentrations [133], suggesting that they are not biocompatible as of yet.

### 5.3. Nanoparticles for Gluing Hydrogels

Conventional adhesives often consist of polymers, since they both dissipate energy under stress and are flexible enough to establish good contact between surfaces. However, it is difficult to glue together hydrogels using polymers, which makes the assembly of gels or tissues very challenging. Inspired by the mechanism of nanocomposite hydrogel formation, Rose and co-authors glued PDMAm hydrogels and liver tissue using silica NPs, in a process named (nano)bridging, where NPs can adsorb to the polymer network or macromolecules in the tissue and act as connectors between the two surfaces [67,73]. If the NPs have diameters comparable to the network mesh size, multiple polymer chains can be absorbed to the same particle, creating an adhesive layer, with the polymers in the gel acting as bridges between NPs. When the adhesion site is strained, some monomers detach from the NPs, thus releasing the tension. These detached segments can easily reattach, or a neighboring strand can be adsorbed to replace the detached segments. This effect leads to an adhesive junction that can withstand high tensions without fracturing. In fact, the adhesion site was stronger than the gel network itself, so fracturing did not occur at the adhesion site [67].

The position of the NP between the two gels or tissues is found to be dependent on elastic and capillary forces, which in turn varies with NP size and the rigidity of the gel or tissue [72]. It is suggested, using a combination of molecular dynamics simulations and theoretical calculations, that nanobridging is enhanced when using large NPs on hard gels. More specifically, the size of the NPs should be large enough to avoid penetration into the network and diffusion from the interface [72].

### 5.4. Hydrogels for Improved Nanoparticle Performance

Many of the applications of nanocomposite hydrogels are based on the improved functionalities of the hydrogels due to the presence of the NPs. However, some recent studies have instead explored hydrogels as a mean to improve the properties and functionalities of NPs. Of special interest here are the ultrathin 2D nanomaterials, NSs.

As recently reviewed [61], the discovery of mechanically exfoliated graphene [90] has led to an enormous research effort in the fields of condensed matter physics, material science, and nanotechnology, fueled by the exceptional properties of these 2D nanomaterials. One of the characteristics that makes these materials unique, the very large specific surface area, makes them attractive to applications in catalysis and sensing. A challenge that is often faced in these systems is NSs aggregation and poor solution stability, induced by the relatively strong van der Waals interactions between the NSs. In fact, these forces have been explored for the assembly of van der Waals heterostructures, where NSs with different compositions are layered to form novel materials [134,135].

When used as fillers, the poor solution stability of the nanomaterials will affect the mechanical performance of the hydrogels, as discussed above. When the application is related to the large surface area of the materials, which is especially appealing in applications within catalysis and sensing, the aggregation of the NSs will necessarily result in a decrease in the efficiency of the system. We will here mention work performed with g-CN as an illustrative example. The g-CN group of materials has gathered significant interest in the past years due to its photocatalytic activity in the visible range. Besides its good light harvesting and catalytic properties, g-CN is relatively easy and cheap to synthesize, and much research has been conducted for tuning the properties of this class of materials. The two-dimensional structure of g-CN facilitates the hybridization with other components, e.g., graphene, and other modifications such as metal doping and sensitization with organic dyes have been attempted, according to the desired application [136]. g-CNs are particularly appealing for environmental applications involving air purification, water disinfection and decontamination, and waste remediation (see ref. [136] and references within). The ideal performance relies on the adsorption of the hazardous pollutants by the large surface area of the NSs and the photo-degradation of the pollutant, which allows the reutilization of the photocatalyst. Jiang and co-authors have recently described the synthesis of a polyaniline/g-CN composite hydrogel for application in organic pollutant removal [96]. Such a system, as opposed to the more traditional structures of the catalysts (tubes, sheets, and wires), allows for a larger accessibility of the surface area and concomitant facilitated adsorption and concentration of the pollutant (methylene blue) into the hydrogel, followed by the in situ oxidization of the pollutant via photocatalysis. In addition to enhancing the accessibility of the NSs to the organic dyes, and thus the photocatalytic performance of the NSs, due to the improved dispersion of the NSs within the polymer network, the separation from the media and reuse of such photoactive hydrogels should be more easily achieved than the nanometer-sized g-CN material on its own. Another advantage of using hydrogels as a matrix for NPs dispersion is the possibility to mold the hydrogels to the shape that best suits a particular application, as demonstrated using PDMAm/g-CN hydrogel nanocomposites [63]. In the same work, the selectivity of the hydrogel nanocomposite to different pollutants was tested, with the capture of positively charged dyes being particularly efficient, due to the negatively charged surface groups of g-CN.

Another interesting example of the applications of hydrogel nanocomposites is found in ref. [88], where He and co-authors describe the assembly of g-CN into tailored 3D architectures, for broadband solar wastewater remediation. While the resulting product was an aerogel membrane, thus outside the scope of this review, the described (woodpile) structures were obtained by 3D printing the g-CN NSs (building blocks) mixed with sodium alginate, for stabilizing the g-CNs and achieving a good rheological (shear thinning) behavior for the extrusion (printing) process. Furthermore, after the printing process, the 3D-printed structures were submerged in a Ca^2+^ solution to induce the gelation of the alginate chains and concomitant formation of rigid and robust structures that were, in the last step, dried to form the aerogels. Thus, the formation of highly ordered g-CN/sodium alginate hydrogel nanocomposites was an intermediate step in the formation of these promising materials.

While very exciting [137], the field of 2D materials also brings a number of challenges. The poor stability of NSs in solution and structural decomposition/degradation can potentially be improved using hydrogels as coatings and/or dispersion medium, as well as hybridization with other materials. Common to other mentioned nanomaterials is the challenging task of synthesising materials with the criteria required for industry and commercialization, in terms of production yield, quantity, and quality. This is especially important in ultrathin 2D nanomaterials, where the physico-chemical properties highly depend on their structural features.

## 6. Nanoparticles as Tools for Studying and Monitoring Hydrogel Properties

In addition to functionalities in modulating the connectivity of the polymer part of hydrogels, including NPs in hydrogels can also be used as a means to monitor their properties. Thus, the combination of particularities of the NPs and observation by various means, mainly optical, provides information on changes in equilibrium swelling, local deformation, and elasticity. These approaches require careful selection of the properties and concentration of the NPs to support the selected observation method.

### 6.1. Crystalline Colloidal Arrays in Hydrogels

**Figure 9:**
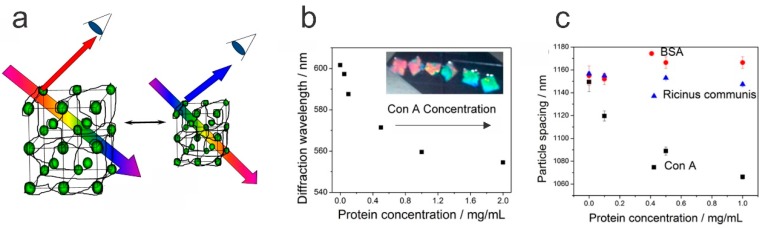
Integration of NP to aid characterization capability of the hydrogels by colloidal crystals embedded in hydrogels. (**a**) The change in the refracted wavelength from 3D crystalline colloidal arrays embedded in hydrogels yields a shift in color when the hydrogel changes its equilibrium swelling state due to various stimuli and molecular details of the responsive network component. Adapted with permission from Cai, Z., et al. Two-Dimensional Photonic Crystal Chemical and Biomolecular Sensors. *Analytical Chemistry* **2015**, *87*, 5013–5025. Copyright (2015) American Chemical Society. (**b**) Changes in diffracted wavelength from a 2D-colloidal crystal mannose hydrogel of AAm, AAc, and mannose side chains as a function of Con A concentration in 0.1 M NaCl aqueous solutions that contain 1 mM Ca^2+^ and 0.5 mM Mn^2+^. (**c**) Calculated colloidal particle spacing for the same 2D colloidal crystal mannose hydrogel as in panel (b) versus the concentration of Con A, Ricinus communis, and BSA. Adapted with permission from Zhang, J.-T., et al. Two-Dimensional Photonic Crystal Sensors for Visual Detection of Lectin Concanavalin A. *Analytical Chemistry* **2014**, *86*, 9036–9041. Copyright (2014) American Chemical Society.

Firstly, monitoring changes in the swelling volume, based on Bragg’s diffraction principle from a lattice of embedded NPs (Figure 9a), requires the inclusion of the NPs in the polymer network with a lattice-like organization. In addition to the crystalline colloid (NP) array (CCA) considered here, inverse opal assemblies and holograms can also be embedded in hydrogels for monitoring their properties [22,138]. The formation of NP arrays embedded in hydrogels to form a sufficiently regular 3D pattern for the Bragg diffraction, exploits electrostatic driven, inter-particle (repulsive) interactions between monodisperse, equally charged NPs. At a sufficiently large concentration, these types of particles adopt periodic nanostructures suitable for characterization by diffraction. The nature of the NPs and the concentration offer a handle to control the particular lattice type, e.g., body centered cubic (BCC) crystals are reported to be favorable using small NPs (diameter < 80 nm) at low concentrations (<3%), whereas face centered cubic lattices are the more prevalent minimum energy organization for larger NPs (diameter > 100 nm) in more concentrated dispersions (>8%), as recently summarized [22]. The integration of the NP lattice in the hydrogel can be realized using procedures as outlined above.

Provided that periodic nanostructures can be realized in the hydrogels, the reflected wavelength from periodically ordered colloids within hydrogels displays a maximum, as obtained from combining the well-known relations of Bragg’s and Snell’s law [139]:

(3) $$\lambda_{\max}=1.633\left(\frac{D}{m}\right){\left(\frac{V}{V_{0}}\right)}^{1/3}\sqrt{n_{a}{}^{2}-\sin^{2}\theta }$$

where *D* is the diameter of the embedded NP, *m* is the order of the Bragg diffraction, and (*V/V_0_*) is the volumetric swelling ratio of the hydrogel. The refractive index *n_a_* in this equation refers to the average of the NP—hydrogel system. This approach provides a quantitative relation between a spectroscopically determined parameter and the extent of swelling as governed by the thermodynamics of the specimen for the actual environmental conditions, as can be deduced from Equation (1).

The application of hydrogel-integrated CCA for the determination of analyte concentration through its effect on the swelling state, and determined based on diffraction from the CCA part, can be grouped into the size of the analyte, e.g., ions, molecules, and macromolecules. CCA embedded in hydrogels for the detection of ions includes, e.g., Pb^2+^ [140,141,142], Hg^2+^ [142,143], and Cd^2+^ [144], which are all examples motivated by the need for the detection of toxic concentrations of heavy metal ions. In addition to the CCA part facilitating the readout process, the hydrogel constructs also need to include recognition moieties with high selectivity and sensitivity to support detection in an interesting analyte concentration range. Concerning the sensitivity and selectivity of the molecular groups incorporated into the hydrogel networks, it is interesting to compare the work by Jana and co-workers [143] exploiting the mercury inhibition of immobilized urease as the recognition and transducing principle with the work by Ye and co-workers [142] that uses an aptamer sequence recognizing mercury. In the former, the authors exploit the de-swelling of a partly anionic polyacrylamide network with the immobilized urease in the presence of urea, with the mechanism of de-swelling being the charge screening of the anionic network due to the product of the enzymatic degradation of urea, ions HCO_3_^−^, and NH_4_^+^. The presence of mercury inhibits the catalytic activity of the enzyme, thus displaying a smaller de-swelling effect. For a NP–hydrogel CCA composite including 360 μmol/mL urease and 0.3 mol/mL carboxylate groups within the hydrogel, a sensitivity of 1 ppb mercury in water was reported, which is below the maximum contamination level stated by EPA, USA, for safe drinking water [23]. Jana and co-workers obtained a similar sensitivity in the recorded wavelength shift for the Hg^2+^ induced swelling (as low as 10 nM) for the aptamer grafted hydrogel. Moreover, they reported that the response was highly selective for Hg^2+^ as compared to other possible ions, among which data were provided for Pb^2+^, Ag^+^, Mn^2+^, Zn^2+^, Mg^2+^, Ca^2+^, Al^3+^, Ba^2+^, Fe^3+^, Cu^2+^, and Cr^3+^. In addition to the aptamer and enzyme strategy, the incorporation of crown ethers in the CCA embedded hydrogels is a widely adopted strategy. This was introduced in the early work of CCA embedded hydrogels by Asher by exploiting a crown ether (4-acryloylaminobenzo-18-crown-6 (AAB18C6)) sensitive to lead, barium, and potassium. The binding of the various ions with specific affinity transforms the crown ether to a charged group, thus inducing swelling of the hydrogel construct. The ionic selectivity and specificity of the crown ethers then underpin the similar properties of the CCA embedded hydrogel swelling properties. The recent review by Lowe and co-workers [23] and references therein provide a more extensive summary of crown ethers and their ionic selectivity as employed as recognition moieties in responsive hydrogels combined with optical readout strategies.

Shifting from CCA hydrogel composites sensitive to ions to materials being able to recognize small molecules, we again find various strategies being employed that integrate and transform the recognition and further processing to a swelling effect readily recorded as shifts in the reflection. Examples of molecules being detected by this approach include, e.g., glucose [145,146], urea [147], ethanol [148], creatinine [149], or ammonia [150]. For glucose, phenylboronic acid (PBA) groups are introduced in the hydrogel network and the CCA embedded hydrogel becomes sensitive to this analyte (and other bearing cis-hydroxyls) in a concentration-dependent manner. Asher demonstrated that this system diffracted light in the visible range, thus ensuring a visible perception of changes in glucose concentrations. A simple observation of a color change in the mirror, provided that such functional material is embedded in a contact lens, and comparison to a color scale calibrated in blood sugar level, is suggested to be beneficial for the health of diabetic patients [145,151]. Later work in this field also includes the improvement of glucose selectivity in the recognition process by modification of the original recognition moiety [146]. While the recognition of glucose induces changes in the swelling of the hydrogel component either by a cross-linking or χ-parameter change of the hydrogel, the specific swelling response to other small molecules can either exploit the same principles, or expand these to, for example, enzymatic or ionic stimuli [22,138].

For CCA embedded in hydrogels designed for the recognition of macromolecules based on, e.g., complementarity in the ssDNA sequence [152], biotin [153], or proteins [154], these groups of materials additionally need to consider transport of the analyte more explicitly compared to the constructs directed towards the smaller analytes. This is due to the general issue on the impact of polymer networks structure (mesh size) on the transport properties of the analytes [155]. Reducing the potential impact of constraining the analyte transport, e.g., by increasing the water content, can inversely affect the mechanical stability of the composite. In the report by Asher and co-workers [154], the reduction in the diffracted wavelength due to reduced proximity between the colloidal particles in the presence of ConA (Figure 9b,c) is driven by the multifunctional binding of mannose to the Con A, thus deswelling the hydrogel in a Con A concentration-dependent manner.

Most of the studies employing CCA within hydrogels explore these constructs as a recognition and transducing element where the hydrogel hosts the recognition functionality by the integration of various functional groups and the transducing principle is based on the associated changes in the equilibrium swelling. The CCA part is embedded to facilitate an optical readout, and also to offer the means for direct perception of changes by the human eye. The approaches mostly focus on the direct correlation between the analyte concentrations and the change in the peak of the wavelength. However, the distribution of the wavelength of the reflected light appears to be much less explored as a basis for understanding hydrogels.

### 6.2. Traction Force Microscopy

While observations of the positional changes within the CCA array embedded in hydrogels are based on the collective behavior of the assumed homogeneous changes in the swelling volume within the illuminated domain, embedding NPs at a lower concentration combined with scattering or optical imaging and subsequent image analysis offer alternative means for the characterization of hydrogels. The image analysis strategy typically establishes trajectories of the embedded particles within the hydrogels, by the simultaneous monitoring of many particles. The incorporation of fluorescent beads facilitates the determination of local deformation of the elastic matrix by connecting sequential positions of the beads from time laps imaging. This information is also conventionally converted to stress fields based on the calibrated elastic modulus of the hydrogel matrix (assuming homogeneous mechanical properties). This group of observations is mostly applied to hydrogels at an equilibrated cross-link density. Typically, the hydrogels are subjected to an applied force, and the approach provides maps of deformation and rate of deformation of the hydrogels. Here, the NP concentration is generally much lower than for the CCA approach, since the initial regular (lattice) organization is not required. Instead, the requirement is that the concentration of the NPs is sufficiently low to allow unique identification of the trajectory of the individual particles between subsequent images.

**Figure 10:**
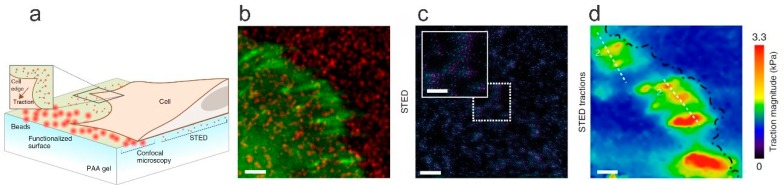
Illustration of traction force microscopy as realized by integrating fluorescent NPs in an elastic matrix (polyacrylamide) and spatio-temporally monitoring their position using either confocal or high resolution (stimulated emission depletion) microscopy to map out deformation and local forces exerted by growing cells on the substrate (**a**) and the process illustrated to map the traction magnitude exerted by growing HeLa cells on an acrylamide gel (**b**–**d**) using STED-Traction Force Microscopy (STFM). The change in the location of the embedded nanoparticles due to the release of traction from the HeLa cells (**c**) and calculated map of traction magnitude on the hydrogel (d) over the same region of interest as shown in (**b**). The scale bar is 2 μm (**b**–**d**). The paxillin in the focal adhesion points of HeLa was labelled with EGFP and 40 nm red fluorescent beads were embedded in the AAm hydrogel (**b**). STED imaging of the positions of the fluorescent beads embedded in the gel under the cell shifted from before (cyan) to after (magenta) treatment with trypsin-EDTA to relax the traction force (**c**) and used as a basis for the analysis of the traction force (**d**). Adapted with permission from Colin-York, H., Eggeling, C.; Fritzsche, M. Dissection of mechanical force in living cells by super-resolved traction force microscopy. *Nature Protocols* **2017**, *12*, 783–796. Copyright (2017) Springer Nature.

In an early example of such a strategy by Dembo and Wang [156], fluorescent latex beads (200 nm) were included at the volume ratio 1:125 relative to a stock solution of 10 wt % acrylamide (AAm) pre-gel used to prepare a covalent anchored AAm gel to a glass surface. The AAm thin film was surface functionalized with collagen or fibronectin [157] and used for determination of deformation as induced by fibroblasts adhering and locomoting on the AAm substrate. The approach enabled the identification of a suggested frontal towing action of the migrating cell, as deduced from changes in traction forces recorded by the deformed substrate preceding observed changes in the direction of the migration. Following this, and earlier reports on the balancing force exerted by a biological entity on an elastic surface [158], the approach of mapping localized positions/forces within a hydrogel include, e.g., characterization in three dimensions [159,160,161] and characterization using super-resolution optical microscopy to reveal dimensional changes at a 50 nm resolution [157,162]. Colin-York and coworkers described the steps involved in realizing high resolution traction force mapping by combining image processing of STED micrographs of a mechanically calibrated hydrogel with embedded nanoparticles [157] (Figure 10). Adherent HeLa cells with fluorescently labelled components in the focal adhesion points (paxillin-EGFP) were grown on a fibronectin coated AAm hydrogel with an elastic modulus of 40 kPa. Analysis of the displacement of embedded fluorescent nanoparticles following the release of the cell anchoring, and thereby the force exerted from the cells on the AAm using methods analogous to particle imaging velocimetry, yields maps of the traction force exerted on the matrix (Figure 10d). The use of STED for the imaging process yields better resolved force maps as compared to the application of conventional confocal imaging. Another recent example is the detailed mapping of human mesenchymal cells inducing local degradation of a 3D network comprised of PEG, including peptide segments susceptible to cell secreted matrix metalloproteinases (MMPs) [163]. The deformation and stress fields in this case displayed a combination of localized stress/deformation changes due to both the displacement of network parts and the cutting of elastically active network chains.

### 6.3. Micro-Rheology

**Figure 11:**
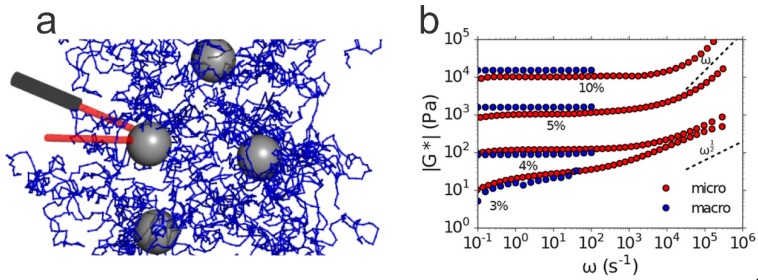
Dynamic light scattering of NPs embedded in hydrogels conducted in backscattering model records information related to the Brownian motion of the NP (**a**), which is further processed to extract rheological signatures of the polymer network. Comparison between micro-rheology data deduced from backscattering mode (micro-rheology) and conventional macroscopic rheology (macro) on cross-linked AAm hydrogels with mass concentrations from 3% to 10% (**b**). The rheological data are presented as the magnitude of the complex shear modulus versus the angular frequency, |G^*^(ω)|. Reprinted with permission from Krajina, B., et al., Dynamic Light Scattering Microrheology Reveals Multiscale Viscoelasticity of Polymer Gels and Precious Biological Materials. *ACS Central Science*, 2017. **3**(12): p. 1294–1303. Copyright (2017) American Chemical Society.

From a hydrogel material perspective, traction force microscopy is devoted to exploitation of the linear, equilibrium elastic properties of the matrices. At variance, NPs embedded in viscoelastic media have, for a long time, been supporting the determination of micro-rheological properties over frequency ranges that, in many cases, also extend the range easily accessible by macro-rheology [164,165,166,167]. There is a range of micro-rheological approaches available, and generally one can group them into passive micro-rheology, where the determination of movement of NPs due to thermal energy forms the basis for extraction of the (localized) rheological moduli, or active micro-rheology, where the probe is actively driven by some external field coupling to the NP, e.g., optical trapping [168,169]. Despite the advocated strongholds of micro-rheology in terms of, e.g., extended frequency range accessible and small sample volume requirements, the main body of applications appears to be within viscoelastic materials possessing terminal relaxations and not that extensively in, e.g., hydrogels displaying (apparent) equilibrium elastic properties on a time scale extending beyond minutes. A couple of examples include the reported nearly frequency-independent complex modulus of AAm hydrogels in the frequency range ω from 0.1 to 100 s^−1^ for polymer concentrations from 10 to 5 wt %, while a stronger frequency dependence was observed below 4 wt % AAm [164] (Figure 11). These features were deduced from data collected in the single scattering limit. Examples of data obtained using this strategy (Figure 11b) illustrate reasonable agreement with bulk rheology data and that data can be collected over an extended frequency range, e.g., exceeding six orders of magnitude, thus providing mechanical spectra with regions containing signatures of various dominating relaxations. Moreover, the small volume requirements for the scattering-based approach as compared to bulk rheology are considered an advantage.

## 7. General Comments and Outlook

Some disadvantages of NP-hydrogel nanocomposites have been pointed out throughout the text, but we will revise them here while pointing out other drawbacks and challenges, as well as an outlook for further developments.

Despite the large interest and potential novel applications of this class of materials, NP-hydrogel nanocomposites still face some challenges before they can be used in real applications. One such challenge is the synthesis of the materials with the criteria required for industry and commercialization. This entails different levels of fabrication, ranging from the synthesis of the nanomaterials and hydrogels to the assembly of the nanocomposites, which also depends on the chosen route for the nanocomposite synthesis. As mentioned, the properties of nanomaterials, particularly the ultrathin 2D NSs, are strongly dependent on the physico-chemical properties of their structural features and the control of such properties for the relatively large-scale synthesis that is required for commercialization is challenging. The controlled preparation of the composites, namely the homogeneous distribution of NPs and NSs in hydrogels (currently achieved by resorting to electrostatics), constitutes another challenge, as well as the formation of anisotropic nanocomposites with controlled structures, or simply the synthesis of micro-gel composites with a defined size and a good batch-to-batch reproducibility. Regarding the latter, flow chemistry, where reactions are performed in channels in a continuous stream rather than in a flask, arises as a promising tool for improving the synthesis of such materials [170]. Regarding macroscopic hydrogel composites, processing techniques such as 3D printing should provide good opportunities for developing hydrogel composites with controlled structures either as final materials or as intermediate steps in the production of aerogels.

The large availability of materials (both polymers and nanomaterials) dictates that the number of possible combinations is enormous, as can be testified by the large amount of publications in the last couple of decades. In many of these, the components are mixed in different proportions and, generally, the mechanical properties of the final material are tested. There is often a lack of discussion on the mechanisms involved in the formation of the hydrogel composite materials and the reason for the improved (or not) mechanical properties. A better understanding of the interactions at play will allow the design of nanocomposite hydrogels with the best properties for the particular applications, whether that entails a more efficient incorporation of the NPs, a better mechanical performance, and a long-term stability of the nanocomposite material; the hydrophilic/hydrophobic nature of the material for improved drug loading capacity and controlled release; or the precise control of shape variations induced by external stimuli, to name a few. Furthermore, the need for optimization of the material for a specific function offers further opportunities for the development of yet new nanomaterials and polymers that can be combined in a range of exciting possibilities. It is thus fundamental to develop new and/or improve existing multi length-scale methodologies for predicting the properties of the materials based on the individual components, as a guide for experimental development. This would allow a more efficient design of materials. Just recently, Lamberti, Barba and coworkers [171] provided an overview of the coupling between solvent diffusion, viscous relaxation time, and a so-called process time, and based on the ranking and differences in these, classified the typical dominant dynamic behavior of hydrogels in terms of viscoelastic and poro-elastic relaxation. Inclusion of NP into the hydrogel can be expected to offer an additional strategy to the network parameters of the hydrogels to tailor-make the dynamic properties of NP-hydrogel composites, which so far, have not been widely explored. Inclusion of high aspect ratio NP in this context may potentially provide strategies for soft materials design with anisotropic dynamic properties.

Another practical aspect to consider when looking into NP-hydrogel composite materials for particular applications is their stability in time regarding durability and fatigue behavior. As reviewed by Creton [18], this is a topic that is seldom discussed within the field of hydrogels, and while the presence of nanomaterials has the potential to improve these properties, these need to be properly assessed. Furthermore, the leaching of nanomaterial from the nanocomposite with time will likely affect the stability and performance of the material, and may also induce environmental toxicity. This may also be the case during the manufacture and disposal of the nanocomposite materials. The cytotoxicity of the material is another practical concern that currently limits the application of such materials in biomedicine and biomimetic materials.

The continuous development of new techniques will allow a better quantitative and predictive knowledge of these materials and the opening of novel and exciting applications.

## 8. Conclusions

NP–hydrogel systems have received much interest in the past decades, fueled by recent suggested applications. Several different ways have been proposed to synthesize nanocomposite hydrogels, ranging from adding NPs to existing hydrogel networks to simultaneously synthesizing NPs and hydrogel in a one pot reaction. It is also possible to coat the NPs with nanogels, which have been explored for, for example, drug delivery applications.

The formation of nanocomposite hydrogels relies on non-covalent attractive interactions between the NPs and the macromolecule network. Thus, an understanding of the intermolecular interactions at play between the surface of the NPs and the macromolecular network is of paramount importance in the development of systems with desirable qualities. The interactions between NPs and polymers can be electrostatic (if charged groups are present) or based on van der Waals forces. The hydrophobic effect may play a significant role in the adsorption of polymers to surfaces, if the monomers are, e.g., methylated. Many studies suggest that hydrogen bonding is the driving force for association, but this might not be very relevant for hydrogels in water.

The individual interactions between NPs and monomers do not necessarily have to be very strong, but the large number of interaction points can lead to a strong adhesion. Due to the non-covalent nature of the interaction between the polymers and NPs, nanocomposite hydrogels can exhibit self-healing abilities, where fractures or cuts can be healed autonomously by reforming bonds across surfaces. Following this same principle, NPs can be used to glue two hydrogels by forming bonds with the polymers in each network and thus bridging the two surfaces. Furthermore, tension and stress can more easily be dissipated, as the non-covalent bonds can break and reform when strained. Therefore, many nanocomposite hydrogels have superior mechanical properties in comparison to pure hydrogels. Taking the opposite perspective, hydrogels have been used to disperse NSs in aqueous solution and to aid the assembly of aerogels with defined 3D structures and photocatalytic activity. This, in turn, increases the accessibility of the large specific surface area of the 2D materials to diverse solutes, thus improving the performance of the nanomaterial. Overall, the composition and characteristics of both NPs/NSs and polymers are important parameters in the synthesis, physico-chemical properties, and application potential of nanocomposite hydrogels. While there is a large number of available polymers and nanomaterials, and the potential to prepare many more, there is a need for accompanying theoretical and modelling studies, as well as the development of new experimental techniques, for a multiscale characterization of the material and a quantitative and predictive understanding of the structure and behavior. This will hopefully contribute to more efficient synthesis processes with better control of the final product and the concomitant expansion of possible applications.

NPs incorporated in hydrogel networks can be used as tools for monitoring hydrogel properties. For example, swelling equilibrium can be monitored by following Bragg’s diffraction from a lattice of embedded NPs arrays. Also, traction force microscopy can give insight into the forces generated by living matter through mapping of the local response of adherent NP-hydrogel composites. The latter strategy affords the characterization of localized forces, primarily in adhesion processes, that are very challenging to determine using alternative strategies.

## Figures and Tables

**Table 1:** Selected recent studies on self-healing nanocomposite hydrogels with different compositions.

Polymer/Nanoparticle Materials	Self-Healing Test	Self-Healing Efficiency	Reference
Hydroxyapatite/calcium-containing silicate glass (bioactive glass)	Compression tests were performed using a Zwick Roell Z2.5 instrument	Complete recovery of mechanical properties	[126]
Poly(*N*,*N*-dimethylacrylamide) or poly(*N*-isopropylacrylamide)/Hectorite clay	Keeping surfaces in contact at ambient/elevated temperatures	Complete recovery of mechanical properties after 10 h	[110]
Bisphosphonated hyaluronan/calcium phosphate	Keeping surfaces in contact for short time (min 5 s)	Almost complete recovery of mechanical properties (98%)	[74]
Carboxybetaine methacrylamide and 2-hydroxyethyl methacrylate/Laponite clay	Keeping surfaces in contact for 5 min, ambient temperature	Mechanically stable and resistant to handling	[112]
Monomer acrylamide copolymerized with *N*-isopropylacrylamide/hectorite clay	Keeping surfaces in contact for 4 days at 20 °C or 4 h at 80 °C	Possessed ultrahigh extensibility and up to 90% strength recovery	[107]
Sodium polyacrylate polymer particles and hyperbranched bis-MPA polyester-64-hydroxyl/reduced graphene oxide	Contact with gentle pressure for 30 s	Nearly full restoration of the electrical conductivity	[127]
Poly(*N*,*N*-dimethylacrylamide)/graphene oxide-hectorite clay	Keeping the cut surfaces in contact and irradiated with a NIR laser under ambient condition	After healing for 2–3 min, strength recovery of ∼96%	[128]
Poly(acrylic acid)/graphene oxide	Contact at 45 °C for 48 h	Almost full recovery of mechanical properties	[118]
Poly(acrylamide)/exfoliated montmorillonite layers	Gels were dried and reswollen at room temperature, and then merged into a single bar	Fracturing did not occur at interface	[116]
Poly(acrylamide)/Laponite	Contact at 80 °C for 24 h	Healing efficiency to up to 50%	[121]
Poly(aspartamide)(GABA/DOPA/EA)/graphene oxide	Fractured gel pieced held in contact	Healing interface strong enough to be stretched without fracturing	[129]
2-acrylamido-2-methyl propane sulfonic acid and acrylamide/zirconium hydroxide	Contact for 5 min-24 h at room temperature	healing efficiency of up to 86% (in strain efficiency)	[122]
Poly(ethylene glycol)/cellulose nanocrystals	Contact at 90 °C for a varying contact time under nitrogen	Up to 78% healing efficiency	[123]
Poly(acrylic acid)/iron ions and 2,2,6,6-tetramethylpiperidine-1-oxyl radical oxidized cellulose nanofibrils (additional cross-linker)	Contact immediately without applied stress for the prescribed contact time 25 °C	Healing interface strong enough to sustain self-supporting, bending, and lifting weights (350 g)	[111]
linear polyurethane chains/maleimide functionalized graphene oxide NPs	Contact at different temperatures for different times	Up to 99% healing efficiency, complete healing at 120 °C after 10 min	[29]
Poly(acrylamide)/Graphene oxide NPs	Contact for different times and different water content	Healing efficiency of up to 92.3%	[115]
Poly(*N*,*N*-dimethyl acrylamide)-gum arabic/TiO_2_	Contact at different temperatures for different times	Healing interface could withstand various deformations such as bending, tying and subsequent elongation. Gels healed at room temperature broke easily at interface	[124]
Poly (2-acrylamido-2-methyl-1-propanesulfonic acid)/Laponite clay and *N*,*N*′-methylenebis (acrylamide) (additional cross-linker)	Contact at 50 °C for 24 h	Fracturing occurred at healing interface, the original network structure could not be completely recovered	[114]
